# Enhanced Immune Responses in Mice by Combining the Mpox Virus B6R-Protein and Aluminum Hydroxide-CpG Vaccine Adjuvants

**DOI:** 10.3390/vaccines12070776

**Published:** 2024-07-15

**Authors:** Junli Li, Xiaochi Li, Jiaxin Dong, Jiazheng Wei, Xiaonan Guo, Guozhi Wang, Miao Xu, Aihua Zhao

**Affiliations:** 1 Division of Tuberculosis Vaccine and Allergen Products, Institute of Biological Product Control, National Institutes for Food and Drug Control, Beijing 102629, China; lijunli@nifdc.org.cn (J.L.); lixiaochi0928@163.com (X.L.); dongjx000@163.com (J.D.); weijiazheng0604@163.com (J.W.); xnguojlu@163.com (X.G.); tbtestlab@nifdc.org.cn (G.W.); xumiao@nifdc.org.cn (M.X.); 2 Key Laboratory for Quality Research and Evaluation of Biological Products, National Medical Products Administration (NMPA), Beijing 102629, China; 3 Key Laboratory of Research on Quality and Standardization of Biotech Products, National Health Commission (NHC), Beijing 102629, China; 4 College of Life Sciences and Biopharmaceuticals, Shenyang Pharmaceutical University, Shenyang 117004, China

**Keywords:** mpox virus, recombinant protein, adjuvant system, compound adjuvant, adjuvanted vaccine

## Abstract

Novel adjuvants and innovative combinations of adjuvants (Adjuvant Systems) have facilitated the development of enhanced and new vaccines against re-emerging and challenging pathogenic microorganisms. Nonetheless, the efficacy of adjuvants is influenced by various factors, and the same adjuvant may generate entirely different immune responses when paired with different antigens. Herein, we combined the MPXV-B6R antigen with BC02, a novel adjuvant with proprietary technology, to assess its capability to induce both cellular and humoral immunity in mouse models. Mice received two intramuscular injections of B6R-BC02, which resulted in the production of MPXV-specific IgG, IgG1, and IgG2a antibodies. Additionally, it elicited strong MPXV-specific Th1-oriented cellular immunity and persistent effector memory B-cell responses. The advantages of BC02 were further validated, including rapid initiation of the immune response, robust recall memory, and sustained immune response induction. Although the potential of immunized mice to produce serum-neutralizing antibodies against the vaccinia virus requires further improvement, the exceptional performance of BC02 as an adjuvant for the MPXV-B6R antigen has been consistently demonstrated.

## 1. Introduction

Adjuvants have been employed for decades to enhance the immune responses to pathogen antigens [1,2]. Their inclusion in vaccine formulations aims to augment, accelerate, and prolong the specific immune response, thereby achieving the desired effect. Adjuvants can be single-component or multi-component systems, offering benefits such as increased antigen immunogenicity, modification of the immune response type, reduced antigen dosage required for successful immunization, fewer booster doses needed, and improved responses in elderly or immunocompromised individuals. Therefore, interest in adjuvants has been growing rapidly, making them a key focus in vaccine research. From the 1920s to the 1990s, despite global efforts to establish novel adjuvants for human vaccines, only aluminum-based adjuvants were licensed. It was not until 1997 that MF59, an oil-in-water emulsion, was approved in Europe as an adjuvant for inactivated seasonal influenza vaccines [3]. Over the next 30 years, five more adjuvants were approved: AS04 (for Fendrix [4] and Cervarix [5]), AS03 (for Pandemrix and Arepanrix [6]), AS01 (for Shingrix [7] and Mosquirix [8]), CpG 1018 (for Heplisav-B [9]), and Matrix-M [10,11,12]. These approvals diversified the use of adjuvants in human vaccines, marking a significant advancement in the field.

BC02, renowned as the BCG-CpG-DNA compound adjuvant System 02, is a sophisticated adjuvant that combines Al(OH)_3_ with BC01 (BCG-CpG-DNA, DNA fragments with unmethylated CpG motifs from Bacillus Calmette–Guerin, the content of unmethylated CpG motifs ranges from 15.75% to 24.75%, and the relative molecular weight ranges from 3000 to 10,000 base pairs). This second-generation adjuvant system, covered by autonomous intellectual property rights, boasts the ability to integrate all compounds effectively and concurrently induce Th1 and Th2 immune responses [13,14]. Each component synergistically enhances the overall immune response in animal and cell models, capable of activating macrophages to participate in the innate immune response [15,16], as preclinical studies with the varicella-zoster virus gE antigen and human papilloma virus 16 and 18 virus-like particles of human papillomavirus types 16 and 18 have shown [17,18,19]. Currently, as an adjuvant component of the lyophilized recombinant tuberculosis vaccine AEC/BC02, it is undergoing Phase II clinical trials (ClinicalTrials.gov ID: NCT05284812). Based on these findings, we hypothesize that BC02 could also enhance the immune response to antigens derived from mpox (formerly known as monkeypox).

Nevertheless, it is crucial to recognize that the efficacy of adjuvants can differ significantly based on multiple factors, and the same adjuvant may elicit different immune responses when paired with different antigens. Enhanced immune responses achieved with one antigen cannot necessarily be extrapolated to another, as antigens differ in their physical, biological, and immunogenic properties, requiring different forms of adjuvant support. Adjuvant selection should be based on the desired type of immune response and formulated with the antigen to achieve the optimal response with minimal side effects. Herein, we aimed to enhance the immunogenicity of the mpox virus (MPXV) protein B6R by combining it with the BC02 compound adjuvant in mice. This approach intends to provide a promising adjuvant candidate for the ongoing development of a monkeypox vaccine.

## 2. Materials and Methods

### 2.1. Ethical Approval

All experimental protocols were carried out at the Institute for Laboratory Animal Resources of the National Institute of Food and Drug Control (NIFDC) in strict compliance with ethical standards. Approval for animal procedures was obtained from the Institutional Animal Care and Use Committee of NIFDC. All mice were maintained under specific-pathogen-free (SPF) conditions, and were housed in a controlled environment with constant temperature, humidity, and a 12 h light/dark cycle. The animals were allowed a three-day acclimation period prior to immunization. Throughout the study, the mice had unlimited access to water and food and were provided with specialized care by expert staff.

### 2.2. Biological Compounds and Chemicals

The novel biological adjuvant BC01, BC02, and mpox-derived antigens A35R, B6R, and M1R were prepared and preserved in the laboratory. Aluminum hydroxide (Alhydrogel^®^) was obtained from InvivoGen (USA), while PBS, RPMI-1640 medium, 100 × Penicillin–Streptomycin, and FBS were obtained from Gibco (USA). The peptide pools for M1R, B6R, or A35R proteins were synthesized by Suzhou-Qiangyao-Biotechnology (China). Mouse TNF-α, IFN-γ, IL-2, IL-4, IL-6, IL-12, IL-1β, MCP-1, GM-CSF, and IL-23 Cytokine ELISA MAXTM Deluxe Kit, and anti-mouse CD3-APC/Cy7, CD8-PE, CD4-FITC, IL-2-PerCp/Cy5.5, IFN-γ-APC, CD19-APC, and CD27-PE-Cy7 fluorescent antibodies were supplied by BioLegend (USA). Goat Anti-Mouse IgG, IgG1, and IgG2a H&L (HRP) were procured from Alpha Diagnostic (USA). IFN-γ, IL-2, IL-4, and IL-5 ELISPOT kits were obtained from Mabtech (Sweden).

### 2.3. Immunization and Sampling

Female BALB/c mice (6–10 weeks old, weighing 20–22 g) were used in this research. The animals were kept in SPF conditions and randomly assigned to diverse groups. Intramuscular immunizations were administered either twice or three times at intervals of 3 or 18 weeks, depending on the specific vaccine schedule and the group assignment. Each group was inoculated with one-tenth of the vaccine. Each immune sample contained either single or multiple combinations of the following: 5 μg of the target antigen, 10 μg of the BC01 adjuvant, 12.5 μg of the Al(OH)_3_ adjuvant, or the BC02 compound adjuvant (10 μg of BC01 + 12.5 μg of Al(OH)_3_). Blank control and negative control mice were injected with 1× PBS or naked antigen in the same dose. After euthanization, peripheral blood and spleen specimens were collected. Blood samples were clotted at 4 °C over an 8 h period before being centrifuged at 1200× *g* for 10 min. The resulting sera were aliquoted and kept at −80 °C. Under aseptic conditions, spleens were dissected, and splenic lymphocytes were separated through density gradient centrifugation after gentle homogenization. Immunological analyses were promptly conducted on the isolated lymphocytes.

### 2.4. ELISA Assays

IgG, IgG1, and IgG2a antibodies against A35R, B6R, and M1R were measured using ELISA kits. Then, 1 μg/mL of either A35R, B6R, or M1R protein was coated onto a 96-well plate, and then incubated overnight at 4 °C. The plates were first washed with 1× TBST and then blocked with 5% BSA at 37 °C for 2 h. Next, serum samples diluted 1:200 with 5% BSA were added and incubated at 37 °C for another 2 h. After a subsequent wash, horseradish peroxidase (HRP)-conjugated goat anti-mouse IgG (1:1000), IgG1 (1:2500), and IgG2a (1:2500) were introduced and incubated at 37 °C for 60 min. Finally, following another wash, the plates were treated with the substrate tetramethylbenzidine and left in the dark for 15 min. Then, 50 μL hydrochloric acid (2 M) was added to terminate the reaction. Absorbance values were measured using an Infinite 200 PRO microplate reader (TECAN) at 450 nm. According to the kit instructions, the levels of Th1 cytokines (IL-2, IFN-γ, and TNF-α), Th2 cytokines (IL-12, IL-6, and IL-4), inflammatory markers, and chemokines (IL-1β, MCP-1, GM-CSF, and IL-23) in the splenic cell culture supernatant of immunized mice were determined using sandwich ELISA.

### 2.5. Neutralization Assays

Neutralizing antibody titer was assessed using live-virus neutralization assays. The vaccinia virus (VACV) Tian Tan strain served as the viral strain for this assay. Sera were assessed for their neutralizing efficacy against live virus by serially diluting samples threefold, starting at a 1:30 dilution, and mixing them with 4 × 10^3^ TCID50 of VACV. The diluted serum samples were then combined with the virus at an equal volume and incubated at 37 °C with 5% CO_2_. After a 1 h incubation, Vero cells (4 × 10^4^ cells/well) were added to each well and further incubated under the same conditions. Following a 48 h incubation period, cell lysis was conducted, and luciferase activity was measured using the Bright Glo Luciferase Assay System on an EnSight plate reader (PerkinElmer). Neutralizing activity was assessed by quantifying luciferase activity in relative light units (RLU), and the 50% live-virus neutralizing antibody titers were determined.

### 2.6. ELISPOT Assays

The levels of mouse splenocytes releasing IFN-γ, IL-2, IL-4, and IL-5 were detected using ELISPOT assays. In brief, a single-cell suspension was prepared from the spleens of immunized mice at 1 × 10^7^ cells/mL. Then, 50 µL of the cell suspension was plated in duplicate on an ELISPOT plate pre-coated with antibodies. The cells were subsequently exposed to M1R, B6R, or A35R peptide pools at concentrations of 1, 5, or 10 µg each; concanavalin A (0.1 µg, positive control); or culture medium (negative control) for 48 h at 37 °C and 5% CO_2_. The ELISPOT kit was employed to identify cells secreting IFN-γ, IL-2, IL-4, and IL-5, and positive spots were quantified using a CTL-ImmunoSpot^®^ S5 microanalyzer (Cellular Technology, USA).

### 2.7. Flow Cytometry Analysis

Cells from spleens were isolated and subjected to flow cytometry analysis. Briefly, 200 µL of splenocytes (1 × 10^7^ cells/mL) was stimulated with 10 µg/mL peptides for the MPXV-B6R protein and 1 µg/mL of CD28 and CD49d antibodies at 37 °C and 5% CO_2_ for 2 h. Brefeldin A (5 µg/mL) was added and incubated with the cells for an additional 4 h. All incubated and stimulated cells were harvested. The samples were diluted with Fixable Viability Stain 510 in PBS at a ratio of 1:2000 and stained for viability (dead or alive) at 500 μL per test. They were thoroughly mixed and incubated at room temperature, shielded from light, for 15 min. After staining, 1 mL of wash buffer was added to each tube and centrifuged at 300× *g* for 5 min. Subsequently, 100 μL of a fluorescent-labeled antibody mixture (CD3-APC/Cy7, CD4-FITC, CD8-PE, CD19-APC, and CD27-PE-Cy7) was added to each tube for surface labeling and cell staining. The tubes were shaken and the cells were allowed to stain in the dark at room temperature for 20 min. For intracellular staining, following surface staining, 250 μL of Fixation/Permeabilization solution was added to each tube to permeabilize and fix the cells. The tubes were gently mixed and allowed to stand at room temperature in the dark for an additional 20 min. Next, 100 μL of IFN-γ-APC and IL-2-PerCp/Cy5.5 antibody mixture was added to each tube for intracellular factor staining, and the tubes were incubated statically at room temperature in the dark for 30 min. Finally, after washing with cell stain buffer, data were obtained using a FACSAria II flow cytometer and then analyzed using FlowJo 10 software.

### 2.8. Statistical Methods

Statistical analyses were performed using GraphPad Prism v8.0. Differences among multiple groups were evaluated using one-way ANOVA followed by Tukey’s multiple comparison test. Data are expressed as mean ± SE, with statistical significance defined as *p* < 0.05.

## 3. Results

### 3.1. B6R Is More Effective than A35R and M1R in Inducing Cellular Immunity

**Figure 1:**
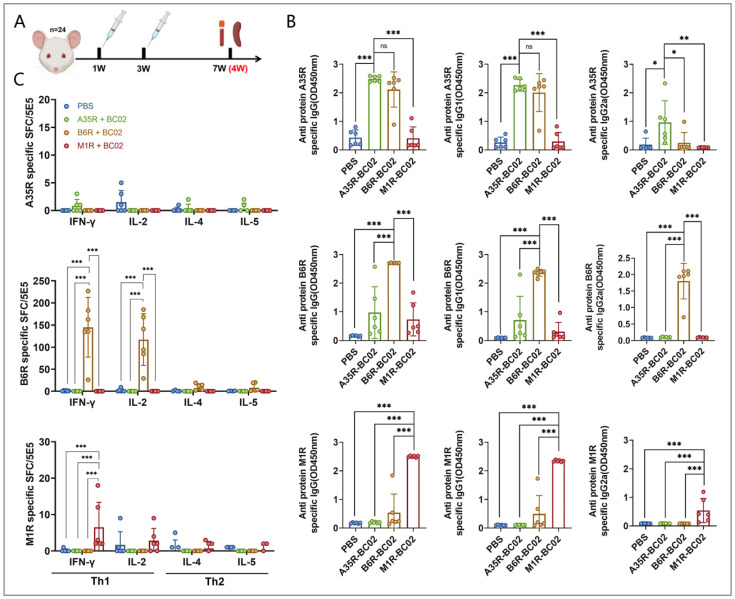
Humoral and cellular immune responses in BC02-adjuvanted MPXV-A35R, B6R, and M1R protein-vaccinated mice were assessed. (**A**) Mice (n = 24, 4 groups with 6 mice per group) underwent immunization with 5 μg BC02-A35R, B6R, or M1R, or were designated as blank controls. Two intramuscular immunizations were administered at 1 week (W) and 3 W. Immunological tests were performed on mice euthanized 4 W after the last immunization. (**B**) The MPXV-specific antibodies IgG, IgG1, and IgG2a against antigens A35R, B6R, and M1R were measured by ELISA. (**C**) The mean numbers of A35R, B6R, or M1R-specific IFN-γ, IL-2, IL-4, and IL-5 spot-forming cells (SFC) in immunized mice were determined by ELISPOT. Data are presented as mean ± SEM. Significance was determined by two-way ANOVA with multiple comparison tests. * *p* < 0.05, ** *p* < 0.01, *** *p* < 0.0001 indicate significant differences; ns indicates no significant difference.

The immunogenic properties of the MPXV-A35R, B6R, and M1R proteins, combined with the BC02 compound adjuvant, were evaluated in mice (Figure 1A). The mice received immunizations with A35R-BC02, B6R-BC02, and M1R-BC02, respectively, via intramuscular injections administered twice, three weeks apart. PBS was used as the blank control group. Four weeks after the final immunization, mouse sera were used to evaluate humoral immunity. The IgG, IgG1, and IgG2a binding antibody responses against the MPXV-A35R, B6R, and M1R proteins were measured using ELISA. Antibodies targeting all three antigens were found in the vaccinated mice, showing a significant increase (Figure 1B).

ELISPOT was used to assess the cellular immunity of mice four weeks after the same dose of booster immunization. The results showed that IFN-γ (Th1) and TNF-α (Th1) were significantly induced in the spleen cells of mice immunized with B6R-BC02. On the contrary, no obvious differences in IL-4 (Th2) and IL-5 (Th2) secretion levels were found between the blank control and the B6R-BC02-vaccinated mice. Notably, neither A35R-BC02 nor M1R-BC02 induced a significant Th-1 or Th-2 immune response (Figure 1C). Based on MPXV antigen-specific ELISA and ELISPOT analyses, the immune response induced by B6R-BC02 was higher than that induced by A35R-BC02 and M1R-BC02. Therefore, the MPXV-B6R protein can be considered a dominant antigen candidate for future adjuvant synergism studies.

### 3.2. BC02 Compound Adjuvant Significantly Enhances Cellular and Humoral Immunity via B6R

After identifying the MPXV target antigen, which is highly effective in inducing cellular and humoral immune responses, we further investigated the enhancement effect of the BC02 compound adjuvant and its components on the immune response to the B6R antigen. It was confirmed that the BC02 adjuvant effectively enhances the immunogenicity of MPXV-B6R, and the synergistic effects among the BC02 compound adjuvant components were analyzed.

**Figure 2:**
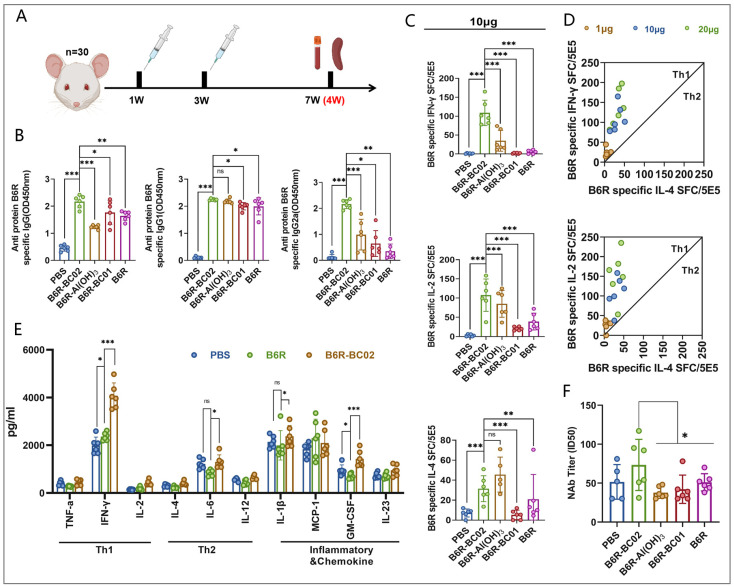
Humoral and cellular immune responses in BC02-, Al(OH)_3_-, and BC01-adjuvanted MPXV-B6R protein-vaccinated mice. (**A**) Mice (n = 30, 5 groups with 6 mice per group) underwent immunization with 5 μg BC02-B6R or were designated blank or negative controls. Two intramuscular immunizations were administered at 1 W and 3 W. Immunological tests were performed on mice euthanized 4 W after the last immunization. (**B**) The MPXV-specific antibodies IgG, IgG1, and IgG2a against antigens A35R, B6R, and M1R were measured by ELISA. (**C**,**D**) The mean numbers of B6R-specific IFN-γ, IL-2, and IL-4 SFC in immunized mice were determined by ELISPOT. (**E**) The supernatant cytokines in spleen cell cultures of mice immunized with different adjuvant vaccines were determined by sandwich ELISA. (**F**) The ID50 was determined by neutralizing antibody assay based on live VACV. Data are presented as mean ± SEM. Significance was determined by two-way ANOVA with multiple comparison tests. * *p* < 0.05, ** *p* < 0.01, *** *p* < 0.0001 indicate significant differences; ns indicates no significant difference.

Mice were immunized with B6R-BC02, B6R-Al(OH)_3_, and B6R-BC01 through two intramuscular administrations at three-week intervals. PBS and the naked B6R antigen served as the blank and negative control groups, respectively. Sera and spleen cells were collected from all mice four weeks after the final immunization to assess the immune response (Figure 2A). Serum antibody analysis showed that the BC02 compound adjuvant and its components, Al(OH)_3_ and BC01, effectively induced the secretion of B6R antigen-specific IgG, IgG1, and IgG2a antibodies in immunized mice. However, the synergistic ability of the BC02 compound adjuvant was significantly better than that of the single adjuvants, particularly in inducing the secretion of IgG2a antibodies (Figure 2B). Unfortunately, only low levels of neutralizing antibodies to the VACV Tian Tan strain were detected in the immunized mice. Although B6R-BC02 produced higher levels than the other groups, the titer of neutralizing antibodies was only slightly above the detection limit (Figure 2F).

In terms of cellular immunity, B6R-BC02 significantly increased the number of B6R-specific IFN-γ, IL-2, and IL-4 spot-forming cells (SFC) in immunized mice, with a notable bias toward inducing a Th1 immune response, regardless of the B6R protein peptide pool stimulation concentration of 1, 10, or 20 μg/mL (Figure 2C,D). Similarly, levels of Th1 (IL-2, IFN-γ, and TNF-α), Th2 (IL-12, IL-6, and IL-4), and inflammation and chemokines (IL-1β, MCP-1, GM-CSF, and IL-23) in spleen cell culture supernatants of different immunized mice were detected by sandwich ELISA. Consistent with the ELISPOT results, the levels of IFN-γ, IL-6, and GM-CSF secreted by the BC02-B6R group were higher compared to the blank control and naked antigen groups, although no obvious differences were detected for the levels of other cytokines (Figure 2E).

### 3.3. BC02-B6R Induces Longer-Term Antibody Responses in Mice

**Figure 3:**
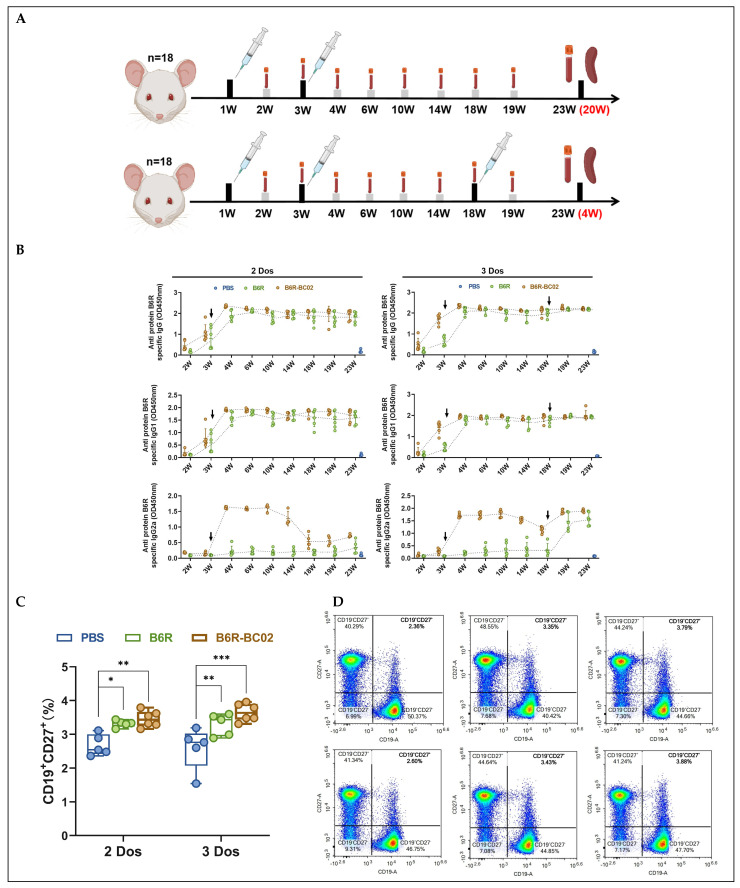
Sustained humoral immune responses in mice inoculated with BC02-B6R were assessed. (**A**) Mice (n = 18, 3 groups with 6 mice per group) underwent immunization with 5 μg BC02-B6R or naked B6R. Two or three intramuscular immunizations were administered at 1, 3, or 18 W, respectively. Immunological tests were performed on mice euthanized 4 W and 19 W after the last immunization. (**B**) The MPXV-specific antibodies IgG, IgG1, and IgG2a against antigens A35R, B6R, and M1R were measured by ELISA. (**C**,**D**) B6R-specific CD19^+^CD27^+^ memory B cells in the spleen were detected by flow cytometry. Data are presented as mean ± SEM. Significance was determined by two-way ANOVA with multiple comparison tests. * *p* < 0.05, ** *p* < 0.01, *** *p* < 0.0001 indicate significant differences.

Although the serum levels of neutralizing antibodies induced by BC02-B6R are low, the long-lasting synergistic nature of the immune process and the rapid induction of an immune response are also important aspects of adjuvant performance. Therefore, we investigated the effects of the BC02 adjuvant on immune persistence and the rapid arousal of the immune response in mice. In this study, mice were inoculated with either B6R-BC02 or naked B6R, receiving a booster immunization three weeks after the primary dose. Serum from all mice was collected periodically to assess the persistence of humoral immune responses. When a decrease in serum antibody levels was detected, a booster immunization was administered with the same dose of the vaccine to evaluate the ability to quickly reawaken an immune response (Figure 3A).

Following the initial immunization, B6R-specific IgG and IgG1 levels in the serum of the B6R-BC02 group increased faster than in the naked B6R group. After the booster immunization, the IgG2a antibody level rapidly increased to a higher level in the B6R-BC02 group, while it did not increase in the naked B6R group. By week 14, after the first booster immunization, serum IgG2a levels began to decline in the B6R-BC02 group, although IgG and IgG1 levels remained elevated. Following the second booster at week 18, the serum IgG2a level in the B6R-BC02 group rapidly returned to its pre-decline level, and the IgG2a level in the naked antigen group also increased significantly (Figure 3B). Flow cytometry data indicated that MPXV-specific memory B cells were markedly elevated in both BC02-B6R- and naked B6R-immunized mice compared to non-immunized controls (Figure 3C,D). This suggests that BC02-B6R immunization exerts an enduring memory B-cell effect.

### 3.4. BC02-B6R Induces Sustained Cellular Immune Response in Mice

**Figure 4:**
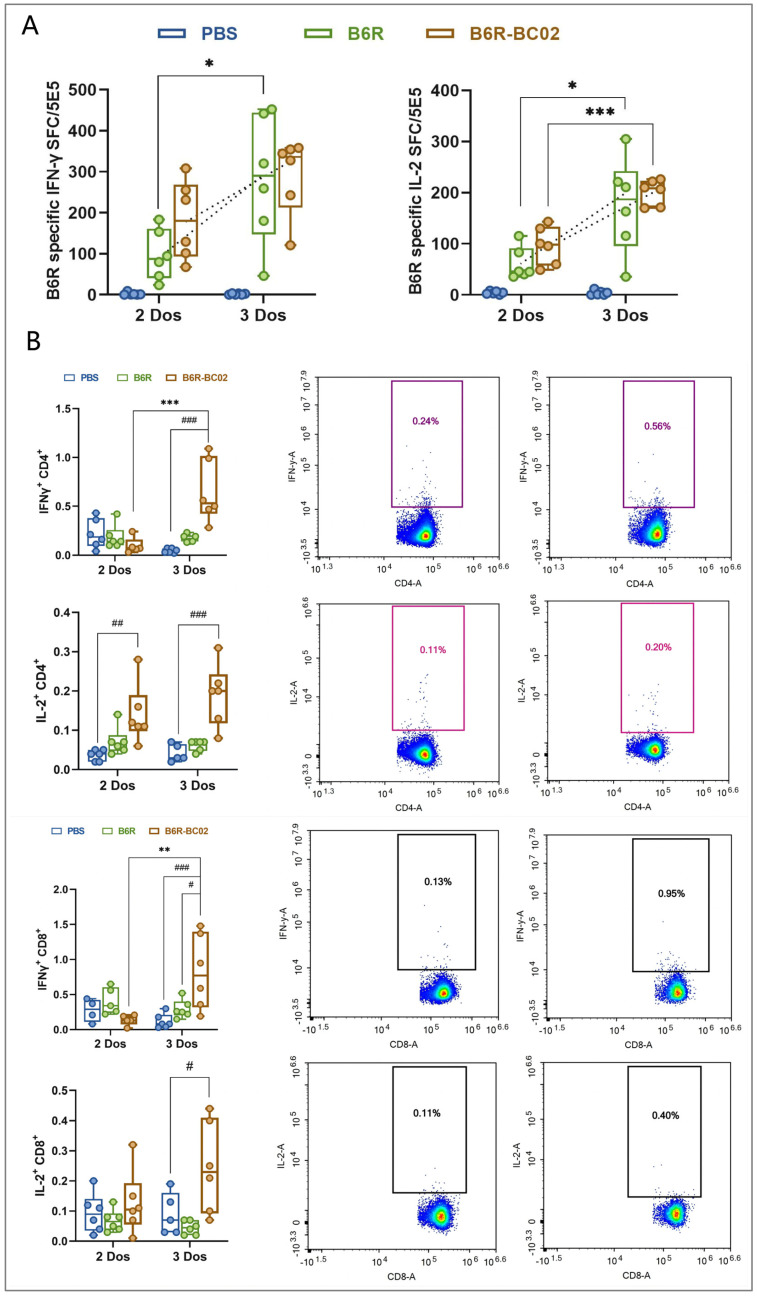
Sustained cellular immune responses in mice inoculated with BC02-B6R. (**A**) The mean numbers of B6R-specific IFN-γ and IL-2 SFC in immunized mice were determined by ELISPOT. (**B**) B6R-specific CD4^+^IFN-γ^+^, CD4^+^LI-2^+^, CD8^+^IFN-γ^+^, and CD8^+^LI-2^+^ T cells in the spleen were detected by flow cytometry. Data are presented as mean ± SEM. Significance was determined by two-way ANOVA with multiple comparison tests. * *p* < 0.05, ** *p* < 0.01, *** *p* < 0.0001 or ^#^ *p* < 0.05, ^##^ *p* < 0.01, ^###^ *p* < 0.0001 indicate significant differences.

In addition to neutralizing antibodies, T cell immunity is also crucial for strong antiviral defenses. We further investigated whether BC02-B6R can induce sustained MPXV-specific T-cell immunity. ELISPOT analysis showed that IFN-γ and IL-2 were significantly induced in the spleen cells of BC02-B6R immunized mice, and this strong response persisted until 20 weeks after the first booster immunization. After the second booster immunization, the level of cellular immune response increased rapidly and significantly (Figure 4A). Flow cytometry results also demonstrated an increase in B6R-specific CD4^+^IL2^+^, CD4^+^IFNγ^+^, CD8^+^IL2^+^, and CD8^+^IFNγ^+^ T cells, with an obvious rise in these populations in the BC02-B6R group following the second booster (Figure 4B).

## 4. Discussion

Currently, three vaccines for mpox, namely, MVA-BN (JYNNEOS^®^, IMVANEX^®^, or IMVAMUNE^®^), ACAM2000, and LC16, have received global approval [20,21,22,23]. These vaccines are derivatives of the smallpox virus vaccine, adapted for use against mpox [24]. It is crucial to note that vaccines based on replication-competent vaccinia virus (VACV) may cause serious side effects, particularly in immunocompromised individuals and those with common skin conditions [25,26,27,28]. Although these vaccines induce neutralizing antibodies against vaccinia virus, their effectiveness against mpox virus is limited [29,30,31,32], potentially leading to breakthrough infections post-vaccination [33,34]. Therefore, finding an equally effective and safe alternative to live virus mpox vaccines remains a priority. In this study, we explored a promising step towards this goal by utilizing purified MPXV A35R, B6R, and M1R proteins produced via a HEK293 cell expression system. These antigens were tested in combination with Al(OH)_3_ and BCG CpG DNA (BC01) as vaccine adjuvants. 

The rational design of recombinant protein vaccines relies on the classical structure of the target virus and the immunogenicity of its proteins. MPXV A35R, B6R, and M1R proteins show significant conservation with orthologous genes from other orthopoxviruses, such as vaccinia virus and smallpox virus [35]. For instance, A35R, similarly to VACV A33R, is expressed on the surface of extracellular enveloped virions (EEV), crucial for viral exit from the endoplasmic reticulum and proper intracellular localization [36,37]. A33R is also targeted by neutralizing antibodies against EEV, particularly in the presence of complement [38]. Additionally, B6R in MPXV is analogous to VACV B5R, essential for wrapping intracellular mature virions (IMV) and inducing actin tail formation on host cells expressing A33 and A36 proteins [39,40]. B5R is a major target of neutralizing antibodies against EEV post-smallpox vaccination [41]. MPXV M1R corresponds to VACV L1R, located on the membrane of IMV. L1R is responsible for viral entry by binding to cell surface molecules and attaching to the viral membrane via a C-terminal transmembrane anchor [42,43,44]. Notably, the N-terminal myristylated domain of L1R is targeted by potent neutralizing antibodies, highlighting its potential as a critical antigenic target [45]. These proteins, along with their counterparts in variola and vaccinia viruses, represent promising targets for developing specific MPXV vaccine candidates due to their importance in generating neutralizing antibody responses [39,46].

Immunogenicity assessments revealed that two doses of A35R-BC02, B6R-BC02, or M1R-BC02 induced robust antibody responses in mice, resulting in high serum levels of MPXV-specific IgG, IgG1, and IgG2a antibodies. Notably, only B6R-BC02 elicited a strong Th1-cell immune response. Based on these findings, B6R was selected as the antigen candidate for further investigation. It was combined with the BC02 compound adjuvant, containing Al(OH)_3_ and BC01 components, for detailed studies on its immunogenicity and efficacy. This study also analyzed the synergistic effects of BC02 adjuvant components and confirmed BC02’s ability to significantly enhance the immunogenicity of MPXV-B6R. Using the same dose and immunization schedule in a mouse model, euthanized mice underwent humoral and cellular immunity tests four weeks after the final immunization. Cellular immunity results demonstrated that B6R-BC02 induced substantial release of B6R-specific IFN-γ and IL-2, favoring Th1 responses significantly over IL-4 induced by B6R-Al(OH)_3_. This distinction is crucial for subunit adjuvant vaccines, as IFN-γ serves as a primary innate mediator post-viral infection. Insufficient IFN-γ levels can heighten susceptibility and severity of infection [47]. Upon maturation, Th1 cells secrete IFN-γ, which activates macrophages and enhances viral attachment to macrophages through B-cell-receptor production [48]. Unfortunately, B6R did not exhibit a clear advantage in stimulating the production of neutralizing antibodies, whether combined with Al(OH)_3_ adjuvant or BC02 compound adjuvant. This suggests that differences in protein structure may influence the antigen’s ability to interact with cells and receptors critical for triggering immune responses [49,50]. The concept of vaccines should evolve beyond whole antigens to focus on epitopes or ligands of immune receptors and their uptake by antigen-presenting cells (APCs). Adjuvants, while enhancing antigen immunogenicity and altering immune response characteristics, cannot fully compensate for antigens lacking inherent immunogenicity or protective immune capabilities.

Despite the suboptimal neutralizing antibody response against VAVR induced by B6R-BC02, the complementary properties of the BC02 complex adjuvant provide sufficient confidence. A single dose of B6R-BC02 established a humoral immune response faster than naked B6R at the same dose. Post-immunization, serum IgG2a antibody levels quickly rose to a higher plateau, indicating BC02’s ability to accelerate memory immune responses. B6R-BC02 also demonstrated robust and rapid immune responses upon detecting reduced levels of specific antibodies, further boosting immunity. Similar results were observed for memory B-cell and specific T-cell immune responses.

In summary, we present the efficacy of a vaccine combining the MPXV-specific antigen B6R with the BC02 compound adjuvant. We compared BC02 and its components Al(OH)_3_ and BC01 in inducing cellular and humoral immunity, confirming BC02’s advantages, such as rapid establishment of initial immune response, strong awakening of memory immune responses, and effective induction of sustained immunity. This underscores that enhanced immune responses achieved with one antigen typically cannot be generalized to another antigen or even the same antigen from a different expression system. While adjuvant performance is crucial, the selection of target antigens emerges as key for successful adjuvant vaccine development.

## Data Availability

The original contributions presented in the study are included in the article, further inquiries can be directed to the corresponding author.
